# Supplementary material for: Modelling and correcting the impact of RF pulses for continuous monitoring of hyperpolarized NMR
Source: Magn Reson (Gott). 2023 Jul 13;4(2):175–86. doi: 10.5194/mr-4-175-2023 (PMC10583294; doi:10.5194/mr-4-175-2023)
Supplement: The supplement related to this article is available online at: https://doi.org/10.5194/mr-4-175-2023-supplement. [file mr-4-175-supplement.pdf]

Supplement of Magn. Reson., 4, 175–186, 2023
https://doi.org/10.5194/mr-4-175-2023-supplement
© Author(s) 2023. CC BY 4.0 License.

Supplement of

Modelling and correcting the impact of RF pulses for continuous monitoring of hyperpolarized NMR

Gevin von Witte et al.

Correspondence to: Sebastian Kozerke (kozerke@biomed.ee.ethz.ch)

The copyright of individual parts of the supplement might differ from the article licence.

This document is structured into three sections: The first two deal with the simulated performance of the three discussed methods to correct for the readout RF pulses. The last section shows experimental (uncorrected) FIDs of both samples to support the signal-to-noise ratio (SNR) discussion of the main text.

For the assessment of the three corrections ($1/\cos^n$, CC-model as introduced in [1] and extended to the steady-state polarization (enhancement) in our work as well as the introduced iterative correction involving the CC-correction) in the simulations, we used a time slicing approach to numerically integrate Eq. 1 of the main paper with RF pulses applied at a fixed repetition time T_R. The simulations included a large noise (compare SNR in the simulations for 2.5° pulses with the experimental FID shown in Figs. S9 and S10 with an SNR of 1000 or more based on the first data point of the FID). The build-ups and decay observed with noise and RF pulses can show very distinct apparent hyperpolarization dynamics compared to the assumed experimental parameters (compare Figs. 1a and S5 as well as Tab. 1 and S1). The discussed corrections were then applied to these apparent build-ups and decays and the results of the corrections can be compared with the expectations based on the assumed experimental parameters.

S1 Simulations of noisy build-ups with RF pulses

The build-ups were simulated based on a time slicing approach of Eq. 1 of the main text. The input parameters (assumed thermal electron polarization, time constant and steady-state polarization) are inspired by experimental values. Application of RF pulses at a fixed repetition time T_R alters the polarization build-up. We applied the extended CC-correction and the iterative correction, involving the time constant estimate of the CC-model, to correct for the impact of the RF pulses. To quantify the performance, we focus on the steady-state polarization and time constant characterizing the build-up. As random noise is applied in the simulation, thousands of random noise configurations are used to estimate the accuracy and precision of the corrections. The resulting histograms for the corrected build-up time together with the expected value are shown in Figs. S1 and S2. The respective results for the corrected SNR are shown in Figs. S3 and S4.

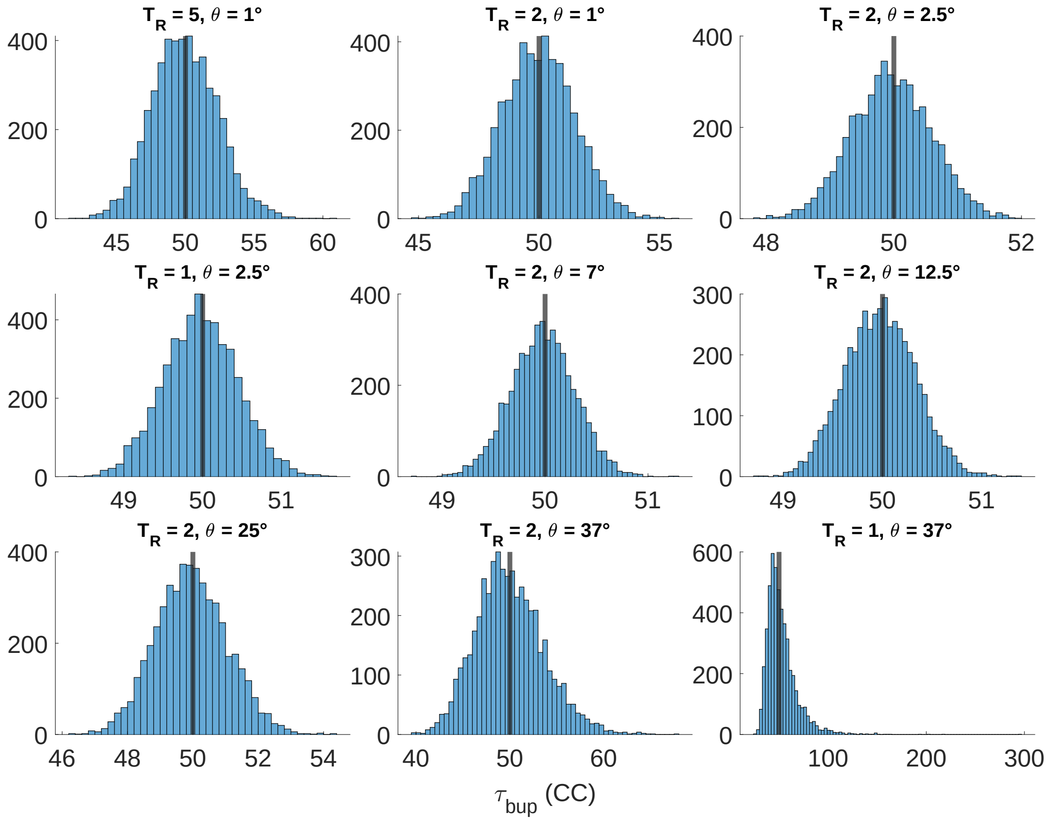

Fig. S1. Results of the CC-correction for the build-up time constant for different flip angles θ and repetition times T_R. The vertical black line refers to the theoretically expected value. Assumed experimental parameters without pulses: $P_0 = 0.3$, $\tau_{bup} = 50$, $A = 1$, noise = $3.2 \cdot 10^{-4}$, 5000 configurations.

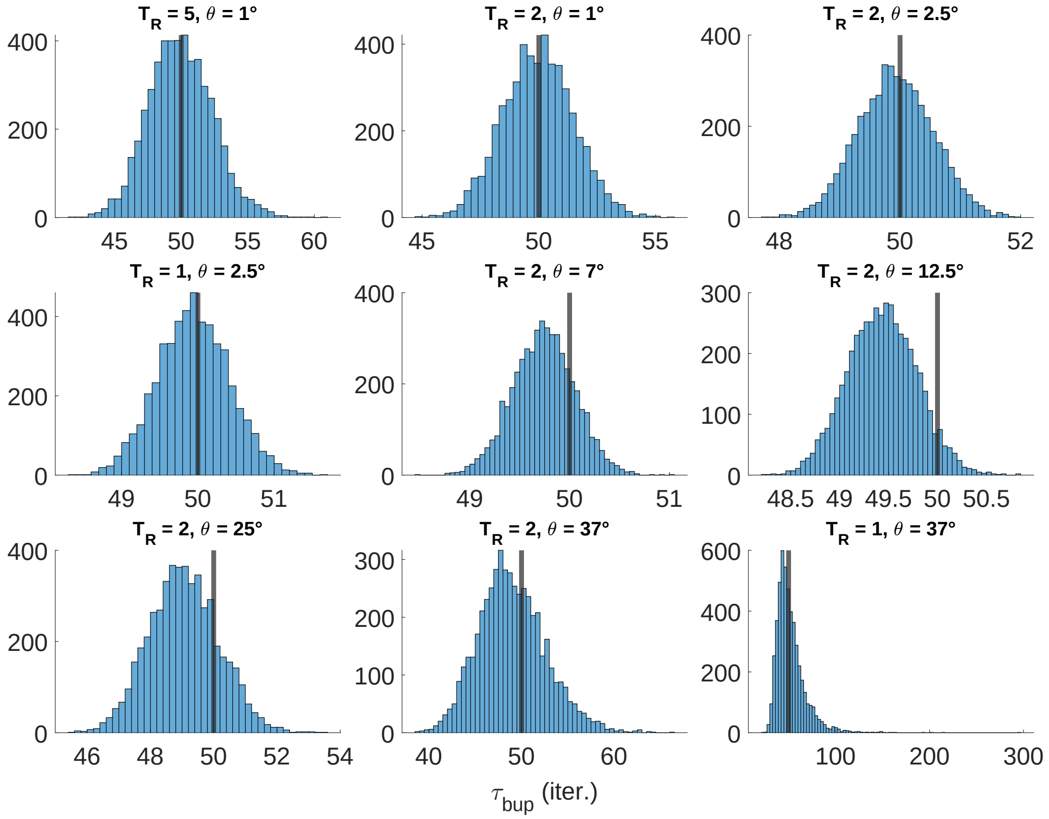

Fig. S2. Results of the iterative correction for the build-up time constant for different flip angles θ and repetition times T_R. The vertical black line refers to the theoretically expected value. Assumed experimental parameters without pulses: $P_0 = 0.3$, $\tau_{bup} = 50$, $A = 1$, noise = $3.2 \cdot 10^{-4}$, 5000 configurations.

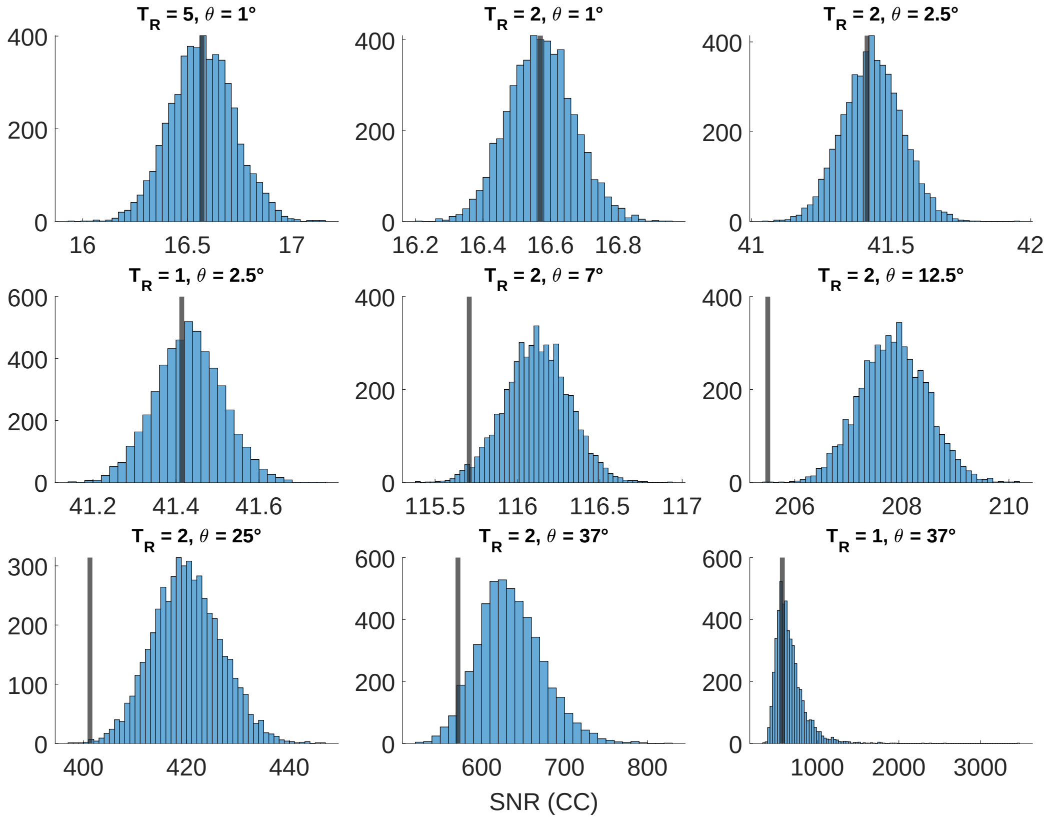

Fig. S3. Results of the CC-correction for the SNR for different flip angles θ and repetition times T_R. The vertical black line refers to the theoretically expected value. Assumed experimental parameters without pulses: $P_0 = 0.3$, $\tau_{bup} = 50$, $A = 1$, noise = $3.2 \cdot 10^{-4}$, 5000 configurations.

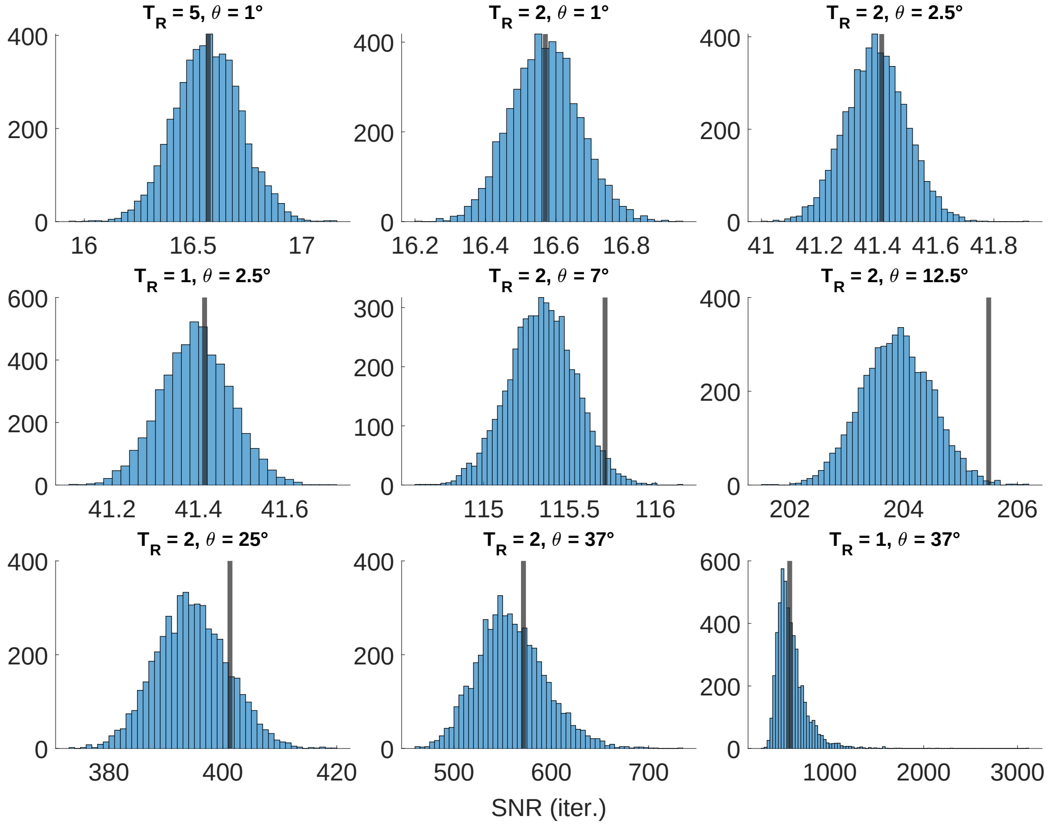

Fig. S4. Results of the iterative correction for the SNR for different flip angles θ and repetition times T_R. The vertical black line refers to the theoretically expected value. Assumed experimental parameters without pulses: $P_0 = 0.3$, $\tau_\mathrm{bup} = 50$, $A = 1$, noise = $3.2 \cdot 10^{-4}$, 5000 configurations.

S2 Simulations of noisy decays with RF pulses

We performed similar simulations for the decay as for the build-up. In particular, the impact of RF pulses in noise-free simulations is studied in Fig. S5 and Tab. S1 - in analogy to Fig. 1 and Tab. 1 of the main text. Subsequently, the effect of noise on the accuracy and precision of the corrections (iterative, CC and $1/\cos\theta^{n-1}$) is studied over thousands of configurations. The resulting histograms are shown in Figs. S6, S7 and S8.

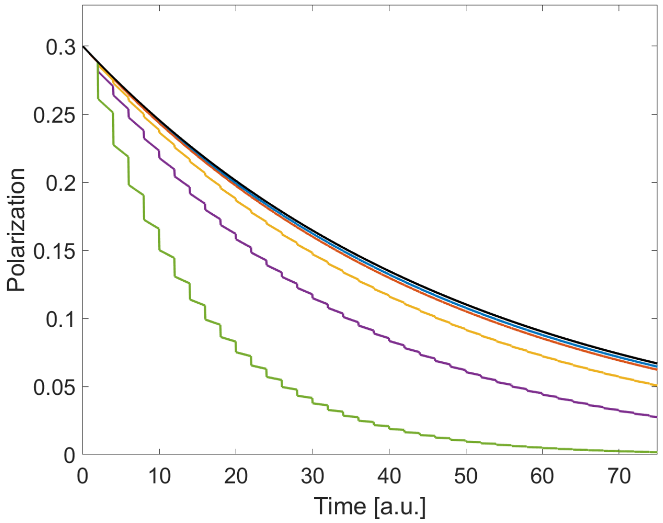

Fig. S5. (Main plot) Comparison of decays under the influence RF pulses without noise. The black curve is without RF pulses. The RF scheme for the other curves (from top to bottom): 2.5°, 2 time units; 2.5°, 1 time unit; 7°, 2 time units; 12.5°, 2 time units; 25°, 2 time units. Assumed experimental parameters without pulses: $P_0 = 0.3$, $\tau_{\text{decay}} = 50$

Flip angle	[°]	2.5	2.5	7	12.5	25
T_R	[a.u.]	2	1	2	2	2
τ_{decay}	[a.u.]	48.8	47.7	42.1	31.2	14.5

Table S1. Fitted decay times of noiseless simulated data under the influence of different RF schemes (compare Fig. S5). Assumed experimental parameters without pulses: $P_0 = 0.3$, $\tau_{\text{decay}} = 50$

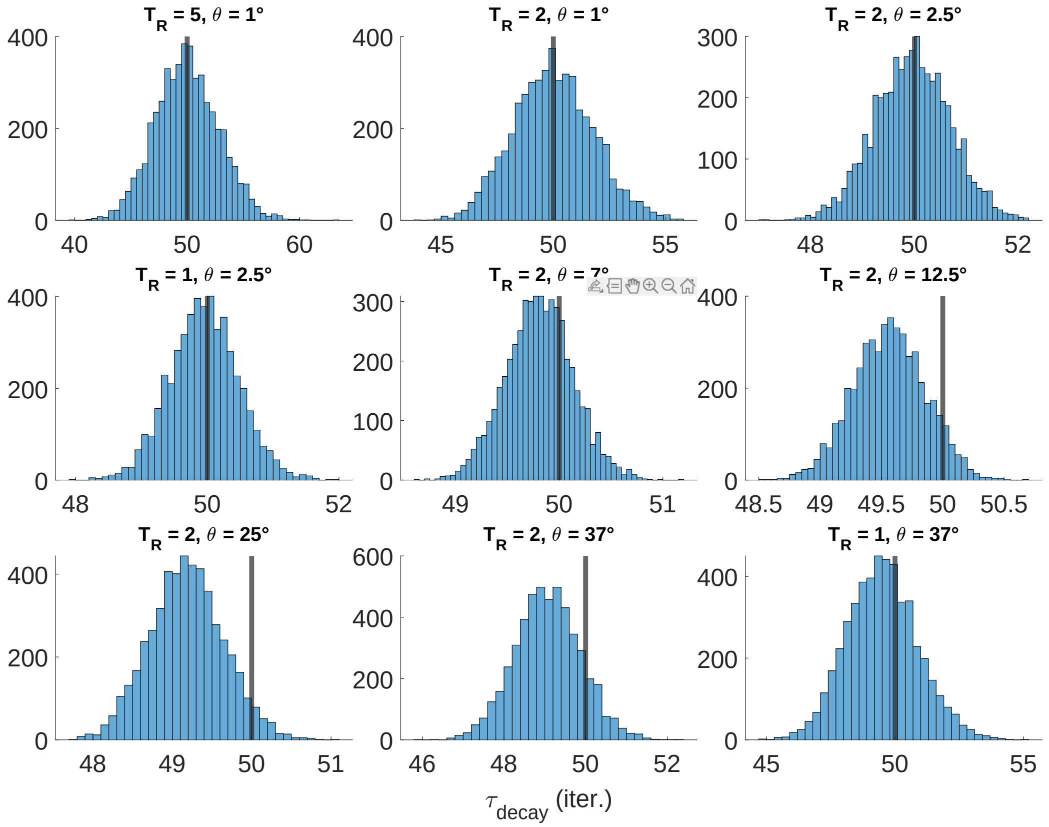

Fig. S6. Results of the iterative correction for the decay time constant for different flip angles θ and repetition times T_R. The vertical black line refers to the theoretically expected value. Assumed experimental parameters without pulses: $P_0 = 0.3$, $\tau_{decay} = 50$, noise = $3.2 \cdot 10^{-4}$, 5000 configurations.

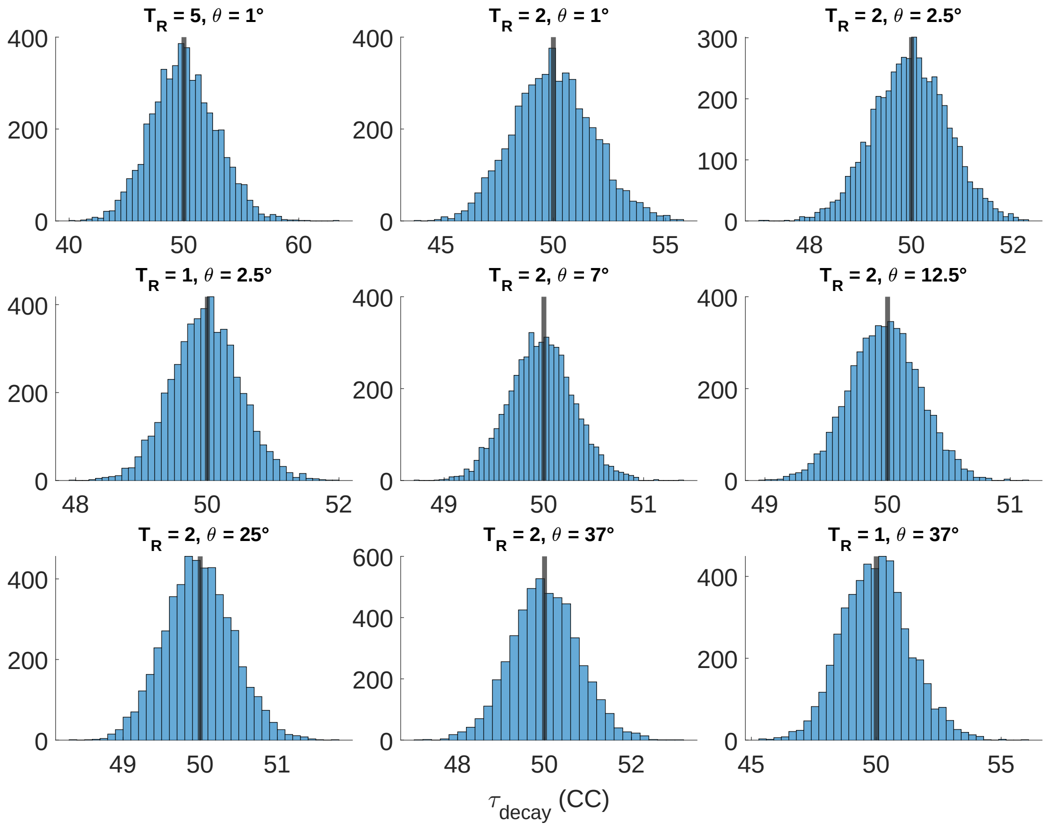

Fig. S7. Results of the CC-correction for the decay time constant for different flip angles θ and repetition times T_R. The vertical black line refers to the theoretically expected value. Assumed experimental parameters without pulses: $P_0 = 0.3$, $\tau_{decay} = 50$, noise = $3.2 \cdot 10^{-4}$, 5000 configurations.

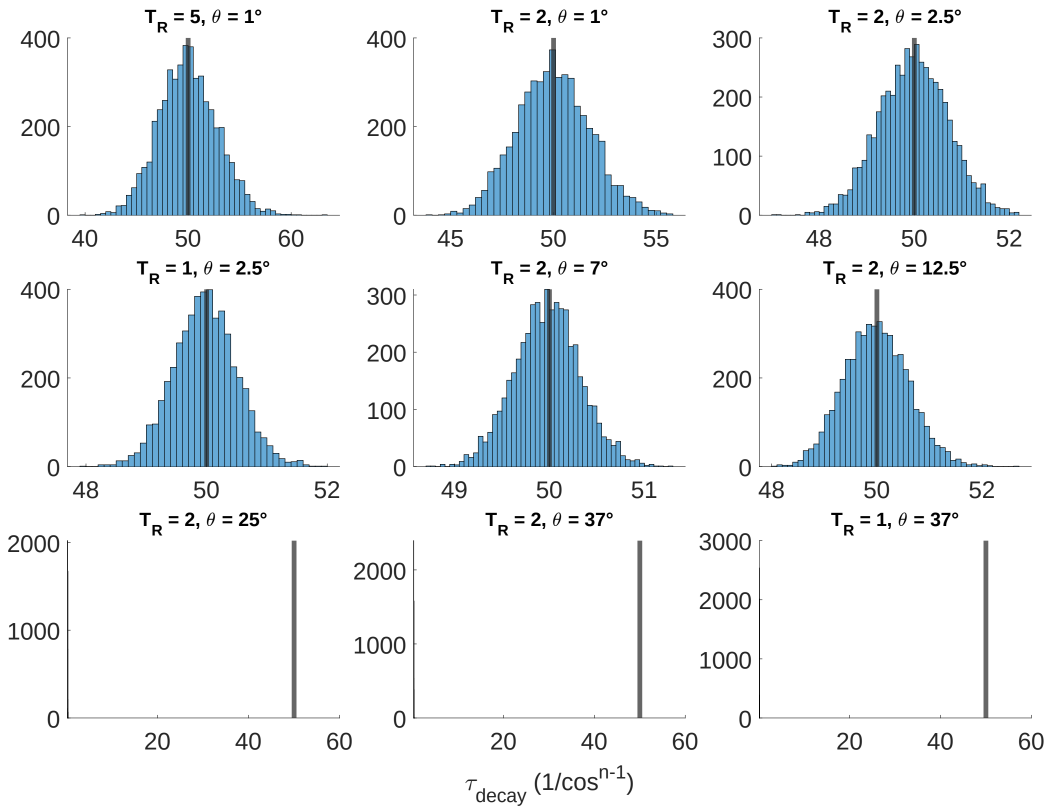

Fig. S8. Results of the $1/\cos\theta^{n-1}$ correction for the decay time constant for different flip angles θ and repetition times T_R. The vertical black line refers to the theoretically expected value. Assumed experimental parameters without pulses: $P_0 = 0.3$, $\tau_{decay} = 50$, noise = $3.2 \cdot 10^{-4}$, 5000 configurations.

S3 Experimental signal

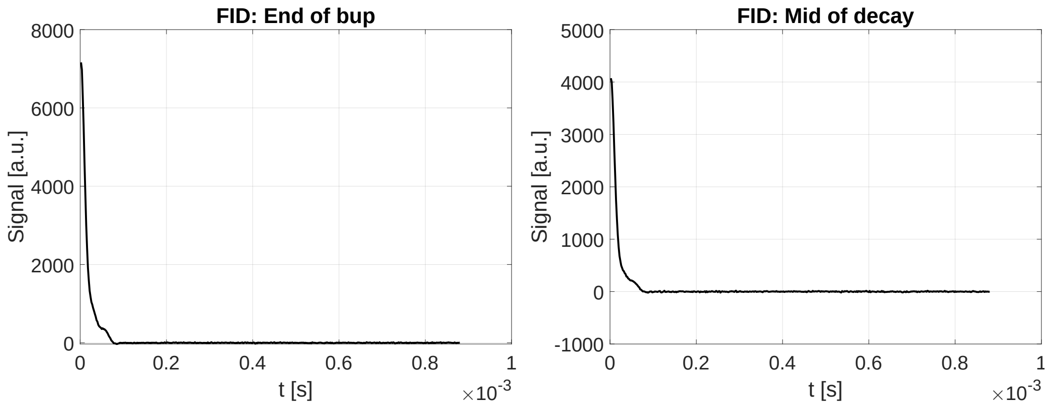

Fig. S9. 2.4°, $T_R = 2$ s experimental (uncorrected) data for 4-oxo-TEMPO in natural abundance water/glycerol $(1/1)_V$. The SNR based on the signal of the first data point compared to the standard deviation of the last 100 points of the FID (no apodization) is 1140 despite a long prescan delay of 18 μs and the short T_2^* due to the high proton concentration.

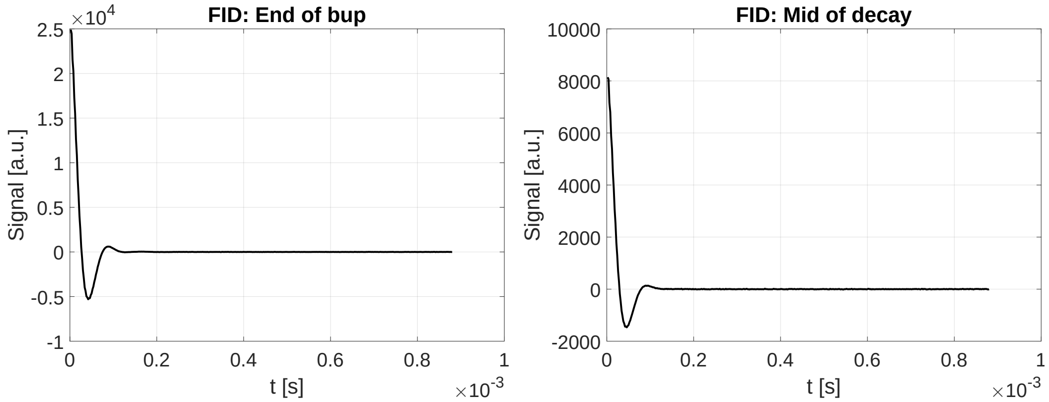

Fig. S10. 2.4°, $T_R = 2$ s experimental (uncorrected) data for 4-oxo-TEMPO in DNP juice. The SNR based on the signal of the first data point compared to the standard deviation of the last 100 points of the FID (no apodization) is 1140 despite a long prescan delay of 18 μs and the short T_2^* due to the high proton concentration.

References

[1] Andrea Capozzi, Tian Cheng, Giovanni Boero, Christophe Roussel, and Arnaud Comment. Thermal annihilation of photo-induced radicals following dynamic nuclear polarization to produce transportable frozen hyperpolarized 13 C-substrates. *Nature Communications*, 8:1–7, 2017.
